# Catastrophic cerebral venous sinus thrombosis secondary to fulminant invasive otomastoiditis

**DOI:** 10.1093/omcr/omaf111

**Published:** 2025-07-27

**Authors:** Nilesh Anand Devanand, Krishnaswamy Sundararajan

**Affiliations:** Royal Adelaide Hospital, Intensive Care Unit, Port Road, Adelaide SA 5000, Australia; Royal Adelaide Hospital, Intensive Care Unit, Port Road, Adelaide SA 5000, Australia

**Keywords:** brain death, cerebral venous sinus thrombosis, otomastoiditis, intensive care unit

## Abstract

A previously healthy, middle-aged immunocompetent man presented to a regional hospital with a 12-h history of right ear pain and discharge following a mild flu-like illness. He rapidly deteriorated neurologically, developing dilated pupils and seizures requiring intubation. Following urgent transfer to a quaternary ICU, multidisciplinary assessment (ENT and Neurosurgery), neuroimaging, and right ear myringotomy confirmed Otomastoiditis with catastrophic cerebral venous sinus thrombosis. CT venography demonstrated extensive thrombosis involving the right sigmoid, transverse, and superior sagittal sinuses. Myringotomy revealed culture-negative blood-stained pus. Pulmonary microbiology results were positive for Influenzae B and the Aspergillus fumigatus complex. Despite maximal medical management, the patient developed bilateral venous infarctions, cerebral edema, and cerebellar tonsillar herniation, progressing to brain death within 48 h. Organ donation proceeded in accordance with his prior wishes. Otomastoiditis can cause rapid, fatal intracranial complications even in healthy individuals, highlighting the need for early imaging, specialist input, and vigilance for neurological decline.

## Introduction

Otomastoiditis, a prevalent disease regardless of immune status, is a rapidly fulminant infection of the mastoid air cells that can spread to the sigmoid and cerebral venous system due to their proximity. The sequential formation of cerebral venous sinus thrombosis produces non-specific clinical symptoms and can be easily missed in the pre-hospital setting. The catastrophic complications of cerebral edema and herniation syndrome are late signs of what should be a treatable and preventable complication.

**Figure 1:**
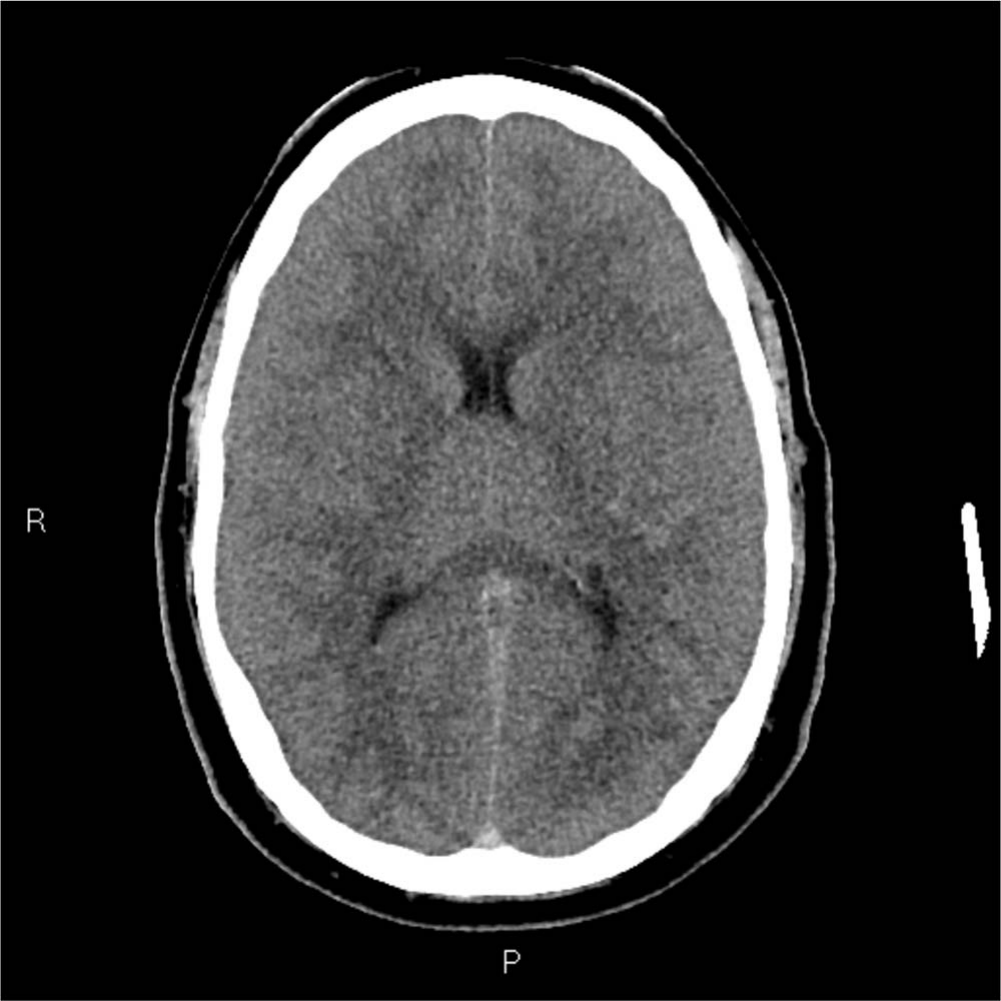
Axial non-contrast CT brain: Classical features of cerebral edema including effaced sulci and gyri, thin slit-like lateral ventricle with altered grey-white differentiation without a mid-line shift.

**Figure 2:**
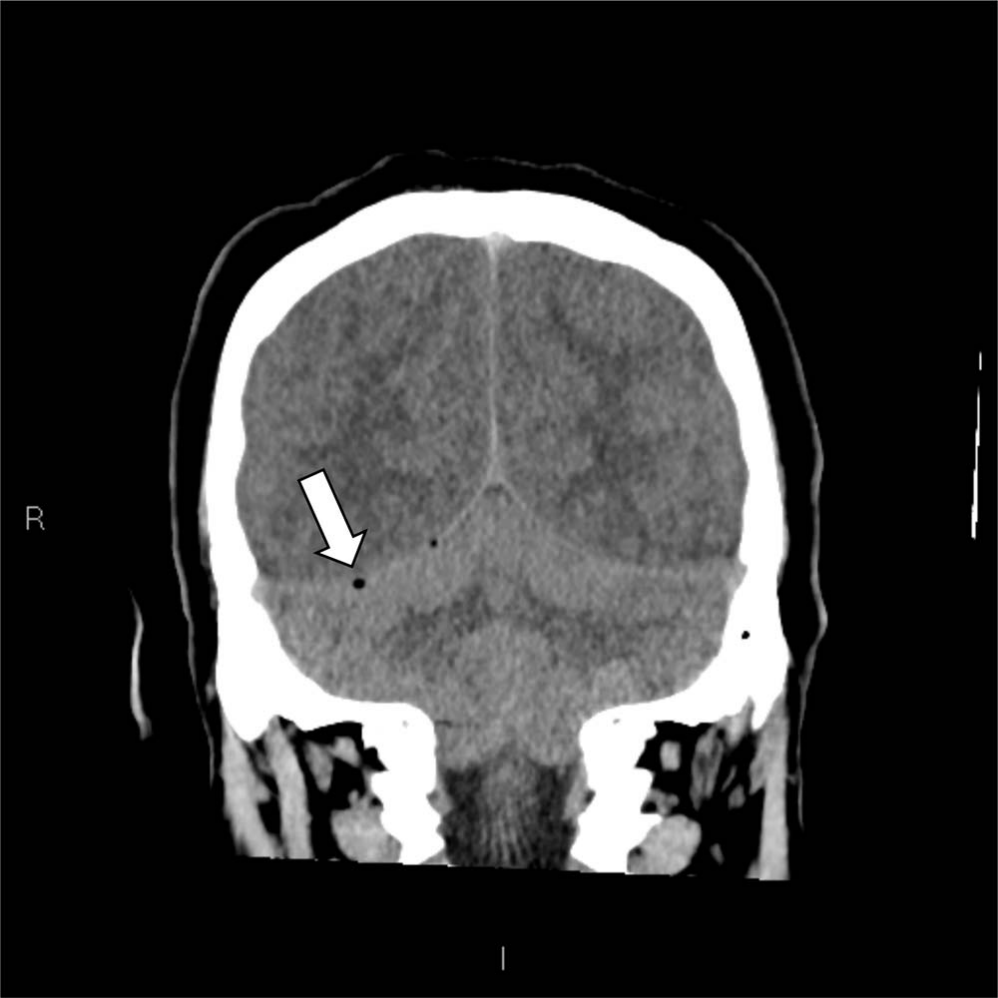
Coronal slice non-contrast CT brain: Cerebellar tonsillar herniation seen along with features of cerebral edema. Note the presence of right-sided pneumocephalus (white arrow) along the tentorium cerebelli.

**Figure 3:**
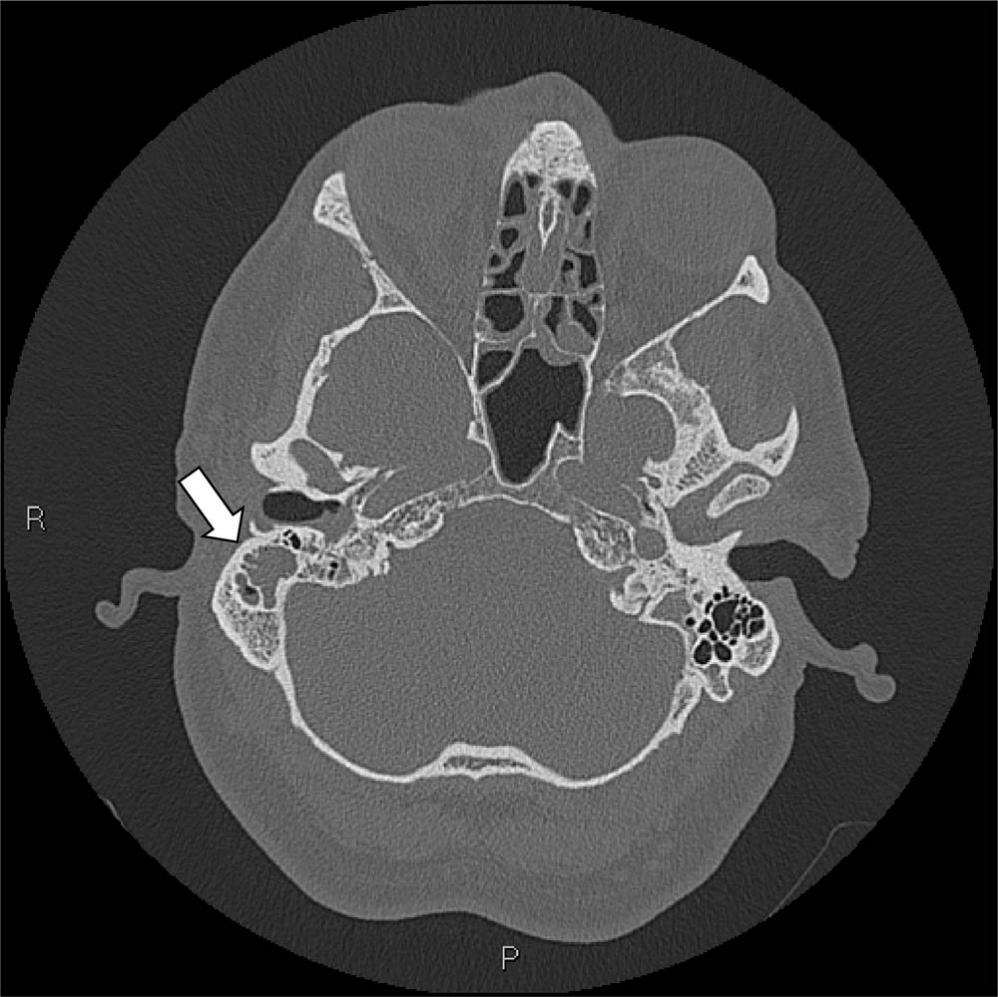
Axial slice CT brain (bone window): Loss of aeration and necrosis within the right-sided mastoid air cells (white arrow), suggesting the presence of fluid-filled cavities from an infective and inflammatory process.

**Figure 4:**
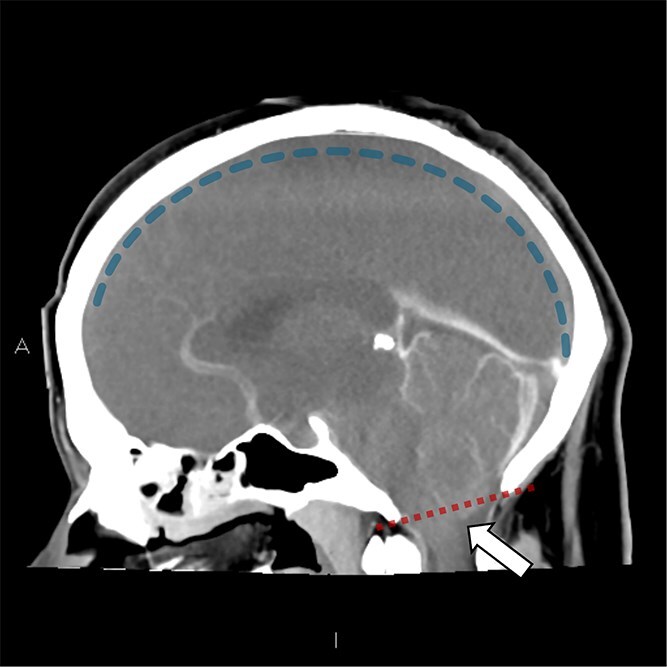
Sagittal slice CT Venogram: Gross cerebral edema with no contrast flow seen within the superior sagittal sinus (blue dashed line) and venous collaterals with early tonsillar herniation (white arrow) indicated by displacement of cerebellum beyond the McRae’s line (radiographic line in red connecting the anterior and posterior margins of the foramen magnum).

**Figure 5:**
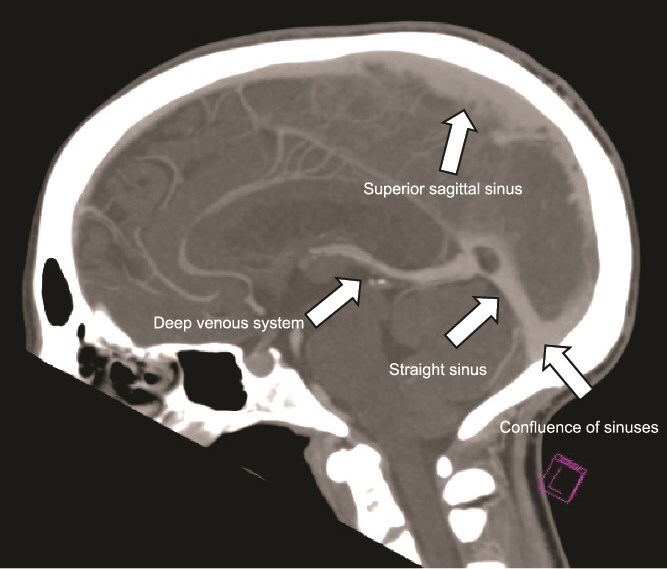
Normal CT Venogram demonstrates contrast within the significant venous sinuses: Sagittal sinus with cortical vein tributaries, transverse and straight sinus, and deep venous system circulation. ^*^(case courtesy of frank Gaillard, Radiopaedia.org, rID: 28100).

**Figure 6:**
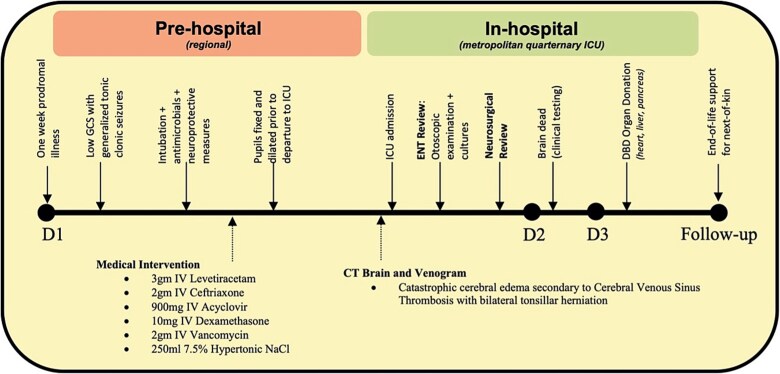
Clinical timeline. GCS: Glasgow coma scale; ICU: Intensive care unit; ENT: Ear, Nose and Throat; DBD: Donation after Brain Death; D1, 2 & 3: Day 1, 2 & 3.

## Case report

A 46-year-old immunocompetent Caucasian gentleman with moderately controlled bronchial asthma presented to a regional hospital with a one-week prodromal mild flu-like illness, followed by 12 h of right ear pain with purulent discharge and severe headache. There is no reported history of prior general practitioner consultation nor a course of antimicrobial therapy for this illness. He was up-to-date with his vaccination status and was HIV-negative.

During the clinical assessment, the patient had a fever and a rapid decline in the Glasgow Coma Scale (GCS) score over 3 h from 15 to 6: Eye 1, Verbal 1, Motor 4. The patient experienced recurrent generalized tonic–clonic seizures necessitating resuscitation and the establishment of a definitive airway. Prior to intubation, his neurological assessment persisted with bilateral withdrawal response (M4) accompanied by dilated, sluggish pupils measuring 6 mm bilaterally. Post intubation, he was loaded with 3gm of Intravenous (IV) Levetiracetam, 2gm of IV Ceftriaxone, 900 mg of IV Acyclovir, 10 mg of Dexamethasone, and 2.5gm of IV Vancomycin. Neuroprotective measures were immediately commenced to avoid secondary brain injury, including maintaining pCO_2_ between 30—35 mmHg. His pupils evolved to become fixed before departure to the Intensive Care Unit (ICU), necessitating 250 ml of 7.5% hypertonic saline with minimal effect.

On arrival in the ICU, the Ear, Nose, and Throat (ENT) team urgently reviewed the patient to perform an otoscopy; they noted right-sided otorrhea consisting of blood and clear fluid with an inflamed intact tympanic membrane and external auditory canal. A right ear myringotomy revealed hemoserous pus discharge with a resultant negative culture. His respiratory viral polymerase chain reaction nucleic acid test was later positive for *Streptococcus pneumoniae* and *Influenzae B*, with a positive endotracheal aspirate for *Aspergillus fumigatus complex*. Given the poor expected prognosis, ENT intervention was limited to bedside myringotomy, and mastoidectomy was deemed non-beneficial.

The plain and contrasted computed tomography (CT) brain scan performed prior revealed that he had cerebral edema, including effacement of the basal cisterns and bilateral downward transtentorial herniation from both of his medial temporal lobes and cerebellar tonsils. The CT venogram revealed no contrast flow in the sagittal, right transverse, and sigmoid sinus. There was pneumocephalus along the falx cerebelli without apparent cerebral abscess.

Input from the neurosurgical team was urgently requested. Upon reviewing the patient and CT images, it was determined that therapeutic options were limited. The minimal hydrocephalus present would not benefit from the insertion of an external ventricular drain. Additionally, after discussions with the interventional radiology team, the extensive thrombosis causing significant absence of cerebral venous flow and collaterals indicated that anticoagulation, clot retrieval, or decompressive craniectomy were unlikely beneficial and did not serve the patient’s best interest. Overnight, he developed progressive cerebral edema and, by the morning, was formally assessed clinically and confirmed to be brain dead. The family was informed and understandably devastated by his rapid deterioration and demise over 48 h. As per his wishes, he was evaluated for and underwent organ donation following brain death, donating his heart, liver, and pancreas.

## Discussion

Cerebral venous sinus thrombosis (CVST) secondary to Otomastoiditis is a life-threatening condition commonly seen in young adults and pediatric age groups [1] with benign symptomatology, frequently leading to late medical presentation and intervention. Despite heterogeneous incidence rates reported in the current literature, the overall annual incidence rate of CVST is estimated to be 8.7 per million [2], with a 3:1 ratio between women and men, and a mean age of 50.9 [3]. Otomastoiditis causing CVST is even more infrequent, with incidences ranging from 3–4 cases per million [3]. Despite a dramatic decrease in clinical incidence [4] in the post-antibiotic era, devastating intracranial complications remain possible, regardless of immune competency.

The pathophysiology involves progressive resorption and demineralization of the ossicles, with subsequent destruction of the tympanic cavity wall and mastoid, inciting the formation of mastoid and cerebral abscesses, meningitis, and sigmoid sinus thrombosis [5]. Common pathogenic etiologies include *S. pneumoniae, Staphylococcus aureus, Streptococcus pyogenes,* and *Haemophilus influenzae*. *Aspergillus* otomycosis, often conferring devastating clinical outcomes, is frequently implicated in immunocompromised patients, with a few isolated case reports in immunocompetent individuals [6].

Treatment is dictated by clinical severity and commonly involves a combination of intravenous antimicrobials complemented by surgical resection of the mastoid. Prognosis varies depending on the presentation timeline, etiology, and intracranial complications, with estimated death and dependence between 10–15% [7].

Proposed mechanisms include thrombosis of cerebral veins, leading to increased venous and capillary pressure, disruption of the blood–brain barrier, and occlusion of the dural sinuses, resulting in decreased cerebrospinal fluid absorption and elevated intracranial pressure [8]. The most frequently involved sites are the transverse sinus (25–60%), followed by the superior sagittal sinus (25–45%) and the straight and sigmoid sinuses (15%); two-thirds of patients have more than one sinus involved [7].

Urgent imaging is pivotal to diagnosis, with guidelines [9] recommending brain magnetic resonance imaging (MRI) and magnetic resonance venography (MRV) or cranial CT venography if the prior options are not available. Treatment often involves anticoagulation and possibly endovascular therapy in patients with worsening neurologic progression.

This case highlights the importance of early neuroimaging in patients with otologic symptoms and progressive neurological deterioration, regardless of their immune status. A multifaceted approach involving community awareness, combined with early and vigilant clinical suspicion, and prompt neuroimaging in the pre- or intra-hospital setting, may lead to earlier detection and thus mitigate a common clinical presentation from catastrophic outcomes and sequelae in the Intensive Care Unit.
